# Changes in Dpysl2 expression are associated with prenatally stressed rat offspring and susceptibility to schizophrenia in humans

**DOI:** 10.3892/ijmm.2015.2161

**Published:** 2015-04-01

**Authors:** HWAYOUNG LEE, JAESOON JOO, SEONG-SU NAH, JONG WOO KIM, HYUNG-KI KIM, JUN-TACK KWON, HWA-YOUNG LEE, YOUNG OCK KIM, HAK-JAE KIM

**Affiliations:** 1 Department of Clinical Pharmacology, Department of Internal Medicine, College of Medicine, Soonchunhyang University, Cheonan, Republic of Korea; 2 Division of Rheumatology, Department of Internal Medicine, College of Medicine, Soonchunhyang University, Cheonan, Republic of Korea; 3 Department of Neuropsychiatry, School of Medicine, Kyunghee University, Seoul, Republic of Korea; 4 Department of Psychiatry, College of Medicine, Soonchunhyang University, Cheonan, Republic of Korea; 5 Development of Ginseng and Medical Plants Research Institute, Rural Administration, Eumseong, Republic of Korea; 6 Soonchunhyang Medical Research Institute, College of Medicine, Soonchunhyang University, Cheonan, Republic of Korea

**Keywords:** dihydropyrimidinase-like 2, social interaction, single nucleotide polymorphism, prenatal stress

## Abstract

Exposure to stress during critical periods of fetal brain development is an environmental risk factor for the development of schizophrenia in adult offspring. In the present study, a repeated-variable stress paradigm was applied to pregnant rats during the last week of gestation, which is analogous to the second trimester of brain development in humans. Behavioral and proteomic analyses were conducted in prenatally-stressed (PNS) adult offspring and non-stressed (NS) adult controls. In the behavioral tests, grooming behavior in the social interaction test, line-crossing behavior in the open field test, and swimming behavior in the forced swimming test were decreased in the PNS group. Western blot analysis and immunohistochemical analysis revealed that the expression of dihydropyrimidinase-like 2 (Dpysl2) or collapsin response mediator protein 2 (Crmp2) was downregulated in the prefrontal cortex and hippocampus of rats in the PNS group. Subsequently, single-nucleotide polymorphisms (SNPs) of the human dihydropyrimidinase-like 2 (*DPYSL2*) gene were analyzed in a population. Two functional SNPs (rs9886448 in the promoter region and rs2289593 in the exon region) were associated with susceptibility to schizophrenia. The present findings demonstrated that the downregulation of genes such as *Dpysl2* and *Dypsl3* in a rat model of prenatal stress may affect subsequent behavioral changes and that polymorphisms of the *DPYSL2* gene in humans may be associated with the development of schizophrenia. Taken together with previous studies investigating the association between the *DPYSL2* gene and schizophrenia, the present findings may contribute additional evidence regarding developmental theories of the pathophysiology of schizophrenia.

## Introduction

Schizophrenia is a well-known psychiatric disorder with a developmental etiology that is associated with abnormal mental functions and behaviors, including delusions, hallucinations, disorganized thinking, and negative symptoms. Additionally, many patients exhibit concomitant mood symptoms, such as depression and anxiety, which may contribute to the 10% lifetime incidence of suicide in schizophrenic patients (1,2). Prenatal stress (PNS) is among the most frequently reported environmental risk factors for the development of schizophrenia in adults (3–8), and the second trimester of pregnancy in humans seems to be the most vulnerable period for insults (8). Furthermore, a number of animal studies have shown that maternal exposure to stress during gestation elevates glucocorticoids and that this type of stress is associated with various biochemical, physiological, and behavioral changes in offspring, including reduced birth weight, cardiovascular and neuroendocrinological abnormalities, attentional dysfunction, enhanced anxiety-related behaviors, and cognitive deficits (9–18). In studies investigating this connection, pregnant female rats are usually exposed to stressful manipulations during the third week of pregnancy, which, in terms of neural development, is equivalent to the second trimester of human gestation (15–17).

Previous findings have demonstrated that PNS or maternal exposure to glucocorticoids may affect the responsivity of the hypothalamic-pituitary-adrenal (HPA) axis and can induce cognitive deficits in offspring (10,16,17,19–22). Cognitive deficits that result from disrupted hippocampal anatomy and impaired function of the HPA axis are also typically observed in patients with schizophrenia (23,24). In animal studies, PNS results in a reduced number of hippocampal synapses as well as fewer neurons in the brain (25). Moreover, the hippocampi of adolescent and adult male rats exposed to PNS exhibit decreases in dendritic length, spine density, and the number of neurons relative to non-stressed (NS) controls (26,27). PNS also causes variable changes of gene expression in the brains of rats, including in genes associated with neural development, cell differentiation, and neurotransmitter function (16,28,29). The frontal poles of PNS rat brains exhibit significant changes in genes associated with postsynaptic density complexes and vesicle exocytosis machinery, including the N-methyl-D-aspartate (NMDA) receptor 1 and 2A subunits, leucine-rich repeat domain-containing proteins (6), brain-enriched guanylate kinase-associated proteins, synaptosomal-associated proteins (24), synaphin/complexin, and vesicle-associated membrane protein 2 (16). A recent study utilizing microarray-based profiling of the hippocampus and frontal cortex investigated changes in gene expression following PNS and identified changes in genes supporting biological processes and/or signal transduction cascades that underlie glutamatergic and γ-aminobutyric acid (GABA)-ergic neurotransmission, mitogen-activated protein kinase (MAPK) signaling, neurotrophic factor signaling, phosphodiesterase (PDE)/cyclic nucleotide signaling, glycogen synthase kinase 3 (GSK3) signaling, and insulin signaling (28). Furthermore, Mairesse *et al* (29) observed alterations in protein expression in the hippocampi of PNS rats and confirmed changes in proteins, such as the LIM and SH3 protein 1 (Lasp1), prohibitin, fascin and transferrin.

Previous findings such as those have led to the hypothesis that PNS-induced phenomena alter the expression of schizophrenia-associated genes via proteomic mechanisms and that these genes ultimately affect susceptibility to schizophrenia in humans. Thus, in the present study, changes in protein expression were examined in the prefrontal cortex and hippocampus of rats exposed to repeated variable PNS. Furthermore, we investigated whether these genes were associated with susceptibility to schizophrenia in humans using genotyping and functional assays of the polymorphisms.

## Subjects and methods

### Animals and stress paradigm

Previously used, pregnant, Sprague-Dawley female rats were purchased from the Central Laboratory Animal Inc. (Seoul, Korea) and arrived at the animal facility on day 7 of gestation. The rats were housed under standard conditions with a 12:12-h light/dark cycle (lights on at 06:30) with free access to food and water. Animal procedures were performed in accordance with the guidelines for the care and use of laboratory animals of the National Institutes of Health of the US.

Beginning on day 14 of gestation, exposure to PNS was initiated and consisted of: i) restraint in well-ventilated cylindrical Plexiglas restrainers for 1 h, ii) exposure to a cold environment (4°C) for 6 h, iii) overnight food deprivation, iv) 15 min of swim stress in room-temperature water, v) reversal of the light-dark cycle, and/or vi) social stress induced by overcrowded housing conditions during the dark phase of the cycle (15,16). Pregnant dams used as controls remained in the animal room from gestational days 14–21 and were exposed to only normal animal-room husbandry procedures. Following birth, the dams and their pups were left undisturbed in their cages until weaning on postnatal day 23. At this time, male and female offspring were separated and group-housed in cages with one or two same-gender littermates with free access to rat chow and water. The animals were exposed to normal animal room conditions from that point onwards until experimental use on postnatal day 35 (16,17).

### Behavioral measures

Modified behavioral tests, including a social interaction test, the open field test, and the forced swim test, were performed, as previously described (15,30–32). The social interaction test was adapted from previous studies (15,31,32) and was conducted in a clear Plexiglas chamber (77×77×25 cm). The room in which the chamber was located was darkened during testing, and the chamber was illuminated by a single 25 W red light bulb placed ~100 cm above the base of the chamber (subject age, 30 days). The sessions were filmed with a video camera (Samsung, Seoul, Korea) placed 150 cm above the cage. The investigator remained outside the test room during testing, and the test arena was cleaned after each test session. Social interaction partners were same-gender siblings who resided in the same cage after weaning and were of approximately equal body weight (in the few cases in which a same-gender sibling was not available, a playmate from similar conditions was used). Each session lasted for 20 min and was scored in terms of the total duration of social play and the number and types of interactions. Specifically, a rater blind to the treatment conditions scored behaviors as aggressive [fighting (kicking, boxing, and wrestling), aggressive grooming and biting] or non-aggressive (sniffing, following and grooming the partner) based on the video. Experimental and target rats were not used in this paradigm more than once, and the arena was cleaned with 70% ethanol after each trial.

The open field test was used to assess exploratory activity and reactivity to a novel environment. On the test day, subjects were removed from their home cage (subject age, 32 days) and individually placed in the start box (15×15×20 cm) of the open field arena (77×77×25 cm) for 5 min. The apparatus was composed of black Polygal, and no background noise was provided. The investigator exited the room, and the behavior of the subject was recorded. Scoring included central boxes entered, line crossings, runs, rears, grooming, cage sniffs and immobile behavior, as previously described (30,31).

As described by previous studies (30,31) the modified forced swim test was used (subject age, 34 days). The rats were individually lowered into a cylinder (height, 40 cm; diameter, 20 cm) filled with fresh heated tap water (25±2°C). After 5 min, the rat was removed and wiped with a clean towel to remove excess water prior to being returned to its home cage. On the following day, each rat was again placed in the cylinder for 15 min during which time swimming, climbing and immobility behaviors were recorded via video camera and by an observer using a stopwatch. The predominant behaviors were counted every 5 sec. Test scores were recorded and included swim behaviors (horizontal movement throughout the chamber and crossing quadrants), climbing behavior (upward-directed movements up the side of the chamber and jump-ups from the bottom of the chamber), and immobility (no additional activity other than keeping the head above water or tiny whip kicks) (30,31).

### Two-dimensional gel electrophoresis

PNS adult offspring and NS adult controls were sacrificed, and their brains were dissected to yield prefrontal cortical and hippocampal tissues. The tissues were washed twice with ice-cold phosphate-buffered saline, sonicated in sample-lysis solution, incubated at room temperature for 1 h, and then centrifuged for 1 h at 15,000 x g. The resulting supernatant fraction was then subjected to two-dimensional gel electrophoresis (2DE); 40-*μ*g samples of protein were separated by first-dimension isoelectric focusing using an immobilized dry strip (13 cm, 3-11NL) and, subsequently, electrophoresis using a 12% sodium dodecyl sulfate polyacrylamide gel electrophoresis (SDS-PAGE) gel. The samples were then stained with silver nitrate, and the strips were subjected to isoelectric focusing at 20°C in a Multiphor II electrophoresis unit connected to an EPS 3500 XL power supply (Amersham Biosciences, Uppsala, Sweden). After focusing, the equilibrated strips were placed on polyacrylamide gels (20×24 cm) containing a gradient of 10–16% SDS, and SDS-PAGE was performed using a Hoefer DALT 2D system (Amersham Biosciences) according to the manufacturer’s instructions. Digitized images of the 2DE gels were quantitatively analyzed using PDQuest software (version 7.0; Bio-Rad, Seoul, Korea) according to the manufacturer’s instructions. The intensity of each protein spot was normalized to the total valid spot intensity. The intensities of the corresponding protein spots from the control and PNS model samples were compared, and spots yielding differences of ≥50% between groups were selected for subsequent analysis.

### MALDI-TOF analysis and database search

Peptides were evaporated with an N2 laser at 337 nm using a delayed extraction approach, and protein analyses were performed using an Ettan matrix-assisted laser desorption/ionization time-of-flight (MALDI-TOF) analysis (Amersham Biosciences, Piscataway, NJ, USA). The search program Mascot, developed by the Matrixscience (http://www.matrixscience.com/), was utilized for protein identification using peptide mass fingerprinting, and spectra were calibrated with trypsin auto-digestion ion peak m/z (842.5099, 2211.1046).

### Immunohistochemistry

The rats were deeply anaesthetized with ethyl ether and perfused with 4% paraformaldehyde. Fixed brains were removed, frozen, and cut into 30-*μ*m sections using a sliding microtome. To detect dihydropyrimidinase-like 2 (Dpysl2 and Dpysl3 expression, frozen sections from the rat prefrontal cortex and hippocampus were blocked with horse and donkey serum, incubated with anti-Dpysl2 (1:400; Cell Signaling Technology, Beverly, MA, USA) and Dpysl3 antibody (1:1,000; EMD Millipore Corp., Billerica, MA, USA) and then incubated with a Cy3-conjugated anti-rabbit secondary antibody (1:2,000; Jackson ImmunoResearch Laboratories, Inc., West Grove, PA, USA). Fluorescence images were captured using a confocal laser scanning microscope (FV10-ASW; Olympus, Tokyo, Japan), and image quantification was performed with ImageJ software using a previously described protocol with slight modifications (33). Briefly, the pixel intensities in the enlarged images (×400) were calibrated by setting the display value range (black) to 255 (green). The threshold level of detection was selected by viewing histograms and then adjusted to distinguish the intensity of the signal from that of the non-specific background. The same threshold level was used for all the images to allow for valid comparisons between the control and PNS model images. The intensity of the labeling was determined using 700-pixel boxes randomly placed at different locations on the labeled area, and background intensity was determined using boxes positioned in areas of no signal.

### Western blot analysis

Prefrontal cortical and hippocampal tissues were lysed in radioimmunoprecipitation assay (RIPA) buffer containing protease inhibitors and then centrifuged at 18,341 × g for 10 min at 4°C. To identify Dpysl2 and Dpysl3, 100 and 20 *μ*g, respectively, of the lysed protein were placed on a 10% SDS gel and transferred onto a polyvinylidene difluoride (PVDF) membrane (EMD Millipore Corp.). After blocking with 5% skim milk, the membranes were probed with anti-Dpysl2 (1:200; Cell Signaling Technology, Inc.; Boston, MA, USA), anti-Dpysl3 (1:20,000; EMD Millipore Corp.), and anti-β-actin (Actb; 1:5,000) antibody overnight at 4°C and then with peroxidase-conjugated secondary antibody (1:2,000) (both from Santa Cruz Biotechnology, Inc., Santa Cruz, CA, USA) for 1 h at room temperature. Immunoreactive bands were detected using an Enhanced Chemiluminescence (ECL) kit (Elpis Biotech Inc., Daejeon, Korea), and quantitative measurements of Dpysl2, Dpysl3 and the Actb protein were obtained using ImageJ software. The mean pixel intensities of Dpysl2, Dpysl3 and the Actb protein were measured by positioning a box around the protein band and subtracting the background intensity. The integrated density values were presented as means ± standard error of the mean (SEM) of the individual protein levels normalized to the integrated density value of Actb. Quantitative measurements of Dpysl2, Dpysl3 and Actb were achieved using ImageJ software (http://imagej.nih.gov/ij). The mean pixel intensities of Dpysl2, Dpysl3 and Actb were measured by positioning a box around the protein band and eliminating the background.

### Subjects

This study included 202 patients with schizophrenia (118 male and 84 female) with a mean age of 46.08±11.9 years (mean ± SD) and 317 control subjects (144 male and 173 female) with a mean age of 44.04±7.8 years (mean ± SD) who had no clinical evidence of any psychiatric disorders. Blood samples were obtained from the schizophrenic and control participants at Soonchunhyang University Hospital and Kyung Hee University Hospital. The patients with schizophrenia were diagnosed according to the Diagnostic and Statistical Manual of Mental Disorders IV (DSM-IV) (34) by two well-trained psychiatrists. Control subjects were recruited following determination that they were mentally fit during an examination provided through a general health checkup program. Written informed consent was obtained from each subject.

### Single-nucleotide polymorphism selection

To evaluate the association between the *DPYSL2* gene and schizophrenia, seven single-nucleotide polymorphisms (SNPs) of *DPYSL2* were genotyped in a Korean population. Five of the seven SNPs were previously described in a Japanese population (rs431246, promoter; rs2289593, missense; rs327222, synonymous; rs708621, synonymous; and rs17666, 3′UTR) (35) and two promoter SNPs (rs4872449 and rs9886448) were added using the National Center for Biotechnology Information (NCBI) website (http://www.ensembl.org; www.ncbi.nlm.nih.gov/SNP). Of the SNPs for *DPYSL2*, all coding SNPs (cSNPs) and all SNPs of the promoter region of the gene (~2,000 bp upstream) were initially selected. Of these, SNPs with >5% minor allele frequency (MAF) and >10% heterozygosity and/or genotype frequencies in the Asian population were included.

### Genotyping

DNA was extracted from peripheral blood using a PureHelix Genomic DNA Prep kit (NanoHelix Co., Ltd., Daejeon, Korea) (36), as previously described. The primer sequences and annealing temperatures used for the analysis of each polymorphism are provided in Table I. Each reaction consisted of a single denaturation at 95°C for 5 min, 35 cycles of denaturation at 95°C for 30 sec, annealing at the appropriate primer pair with an annealing temperature for 30 sec, and then an extension at 72°C for 30 sec. A final extension step at 72°C was performed at the end of the program for 10 min. Following amplification, the polymerase chain reaction (PCR) products were digested overnight with the corresponding restriction enzyme (Table I) according to the manufacturer’s instructions. The digestion products were subsequently electrophoresed on 3.0% agarose gels and stained with SYBR-Green (Invitrogen, Carlsbad, CA, USA). One of the seven SNPs (rs9886448) was analyzed using the high-resolution melt (HRM) method of the Eco™ Real-Time PCR System (Illumina, Inc., San Diego, CA, USA). The reaction was initiated with a single denaturation at 95°C for 10 min, followed by 40 three-step cycles of denaturation at 95°C for 10 sec each, annealing at the appropriate primer-pair annealing temperature for 15 sec, and then an extension at 72°C or 15 sec. Subsequently, the PCR products were heated to 95°C for 15 sec and then cooled down to 55°C for 15 sec. The melt curves were then produced by measuring fluorescence during a temperature increase from 55°C to 95°C (with0.1°C increments).

### Statistical analysis

Western blot analysis and immuno histochemical image quantifications were performed using ImageJ software. Groups were compared using Student’s t-test, and P<0.05 was considered to indicate a significant result. The Hardy-Weinberg equilibrium (HWE) was assessed using the SNPstats program (http://bioinfo.iconcologia.net/SNPstats), and Haploview version 4.2 (Daly Lab Inc., Cambridge, MA, USA) was employed to determine the linkage disequilibrium (LD) block. SNPStats was used to evaluate the odds ratios (ORs), 95% confidence intervals (CIs), and P-values. The multiple logistic regression analyses were conducted using age and gender as covariates and Bonferroni’s correction was applied by multiplying the P-values by the number of SNPs (n=7).

## Results

### Social interaction test

Slight differences between the NS and PNS groups were observed (Table II). In particular, one of the non-aggressive behaviors, i.e., that of partner grooming, was significantly decreased in the PNS group (P=0.011)

### The open-field test

NS and PNS offspring were tested in the open field for 20 min. The PNS group had a significantly greater number of line crossings (P=0.014) and showed a trend towards a greater number of entries into the center (P=0.068) (Table III) relative to the NS group.

### The forced swim test

**Figure 1:**
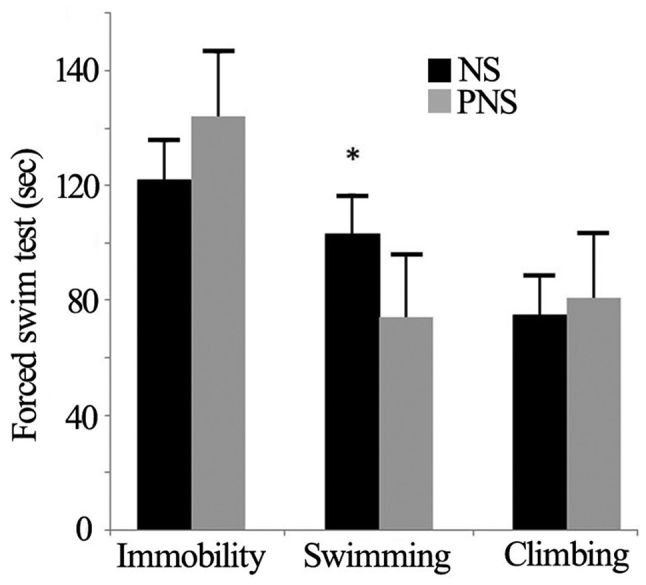
Behavioral response in the forced swim test. Comparison between NS and PNS offspring (n=10 in each group). Marks indicate a decrease in swimming. Data are presented as means ± SEM. ^*^P<0.05. NS, non-prenatal stressed offspring; PNS, prenatal stressed offspring; SEM, standard error of the mean.

In the forced swim test, PNS offspring exhibited fewer immobility behaviors compared with NS rats (P=0.029) (Table IV and Fig. 1). However, the climbing and swimming behaviors of the two groups did not differ (P>0.05).

### Proteomic analysis

**Figure 2:**
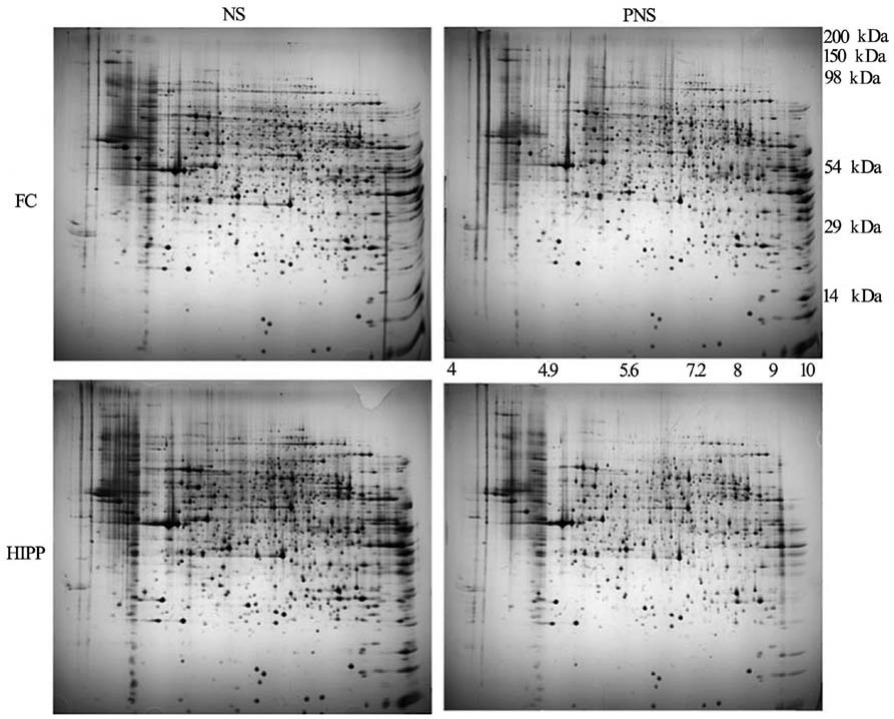
Protein profiles of two-dimensional gel electrophoresis. FC, prefrontal cortex; Hipp, hippocampus; NS, non-prenatal stressed offspring; PNS, prenatal stressed offspring.

To compare protein expression in the prefrontal cortices and hippocampi of PNS and NS rats, the soluble proteins were extracted and evaluated on non-linear pH 3-11 gel strips. From the silver-stained 2D gels, 360 protein spots in the prefrontal cortex and 349 protein spots in the hippo-campus were visualized (Fig. 2). Of these, 31 protein spots in the prefrontal cortex and 30 protein spots in the hippocampus differed significantly according to the MALDI-TOF analyses (95% CI). Compared with the NS group following triplicate runs, the PNS group had five protein spots that were upregulated and 26 spots that were downregulated in the prefrontal cortex (Table V). Additionally, five spots were upregulated in the hippocampus and 30 spots were downregulated in the PNS compared with the control rats (Table VI).

### Confirmation of downregulated proteins in PNS rats

**Figure 3:**
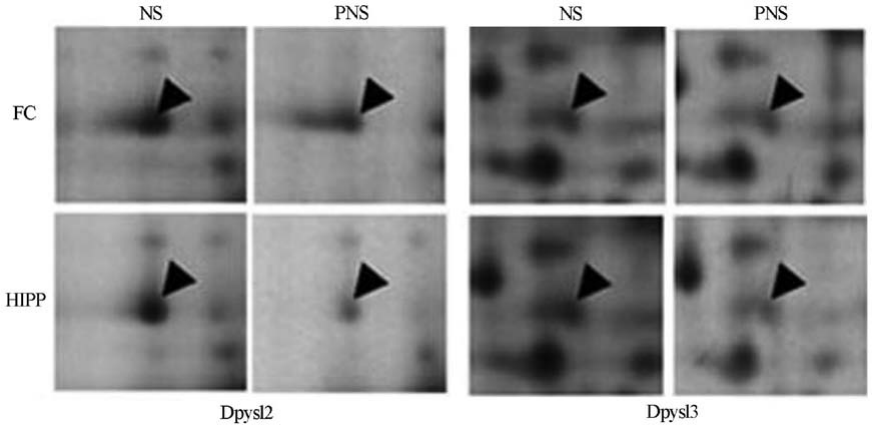
Two-dimensional gel electrophoresis patterns of protein expression in rat brains for two selected proteins. FC, prefrontal cortex; Hipp, hippocampus; NS, non-prenatal stressed offspring; PNS, prenatal stressed offspring. Arrowheads are protein spots, showing different changes among the groups.

**Figure 4:**
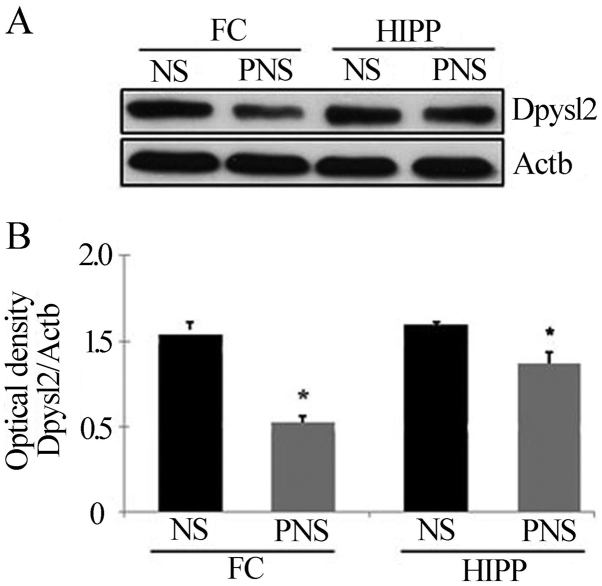
Western blot analysis of Dpysl2 expression in the brains of PNS-induced rats. (A) Dpysl2 expression was detected by western blotting with Actb used as an internal control. PNS rats exhibited decreased Dpysl2 expression in the prefrontal cortex and hippocampus. (B) Quantitative analysis of western blot data for Dpysl2 expression showing a significant difference in Dpysl2 levels in PNS vs. control rat brains (^*^P<0.05 compared with the NS group in the prefrontal cortex and hippocampus). Dpysl2, dihydropyrimidinase-like 2; FC, prefrontal cortex; Hipp, hippocampus; NS, non-prenatal stressed offspring; PNS, prenatal stressed offspring; SD, standard deviation.

**Figure 5:**
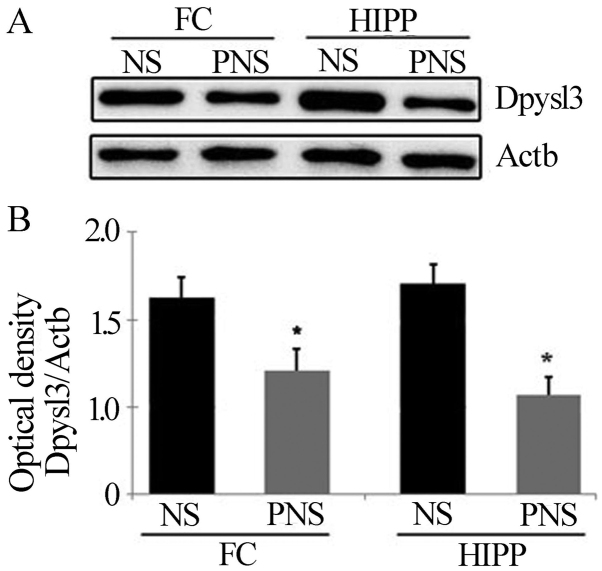
Western blot analysis of Dpysl3 expression in the brains of PNS-induced rats. (A) Dpysl3 expression was detected by western blotting with Actb used as an internal control. PNS rats exhibited decreased Dpysl3 expression in the prefrontal cortex and hippocampus. (B) Quantitative analysis of western blot data for Dpysl3 expression showing a significant difference in Dpysl3 levels in PNS vs. control rat brains (^*^P<0.05 compared with the NS group in the prefrontal cortex and hippocampus). Dpysl3, dihydropyrimidinase-like 3; FC, prefrontal cortex; Hipp, hippocampus; NS, non-prenatal stressed offspring; PNS, prenatal stressed offspring; SD, standard deviation.

**Figure 6:**
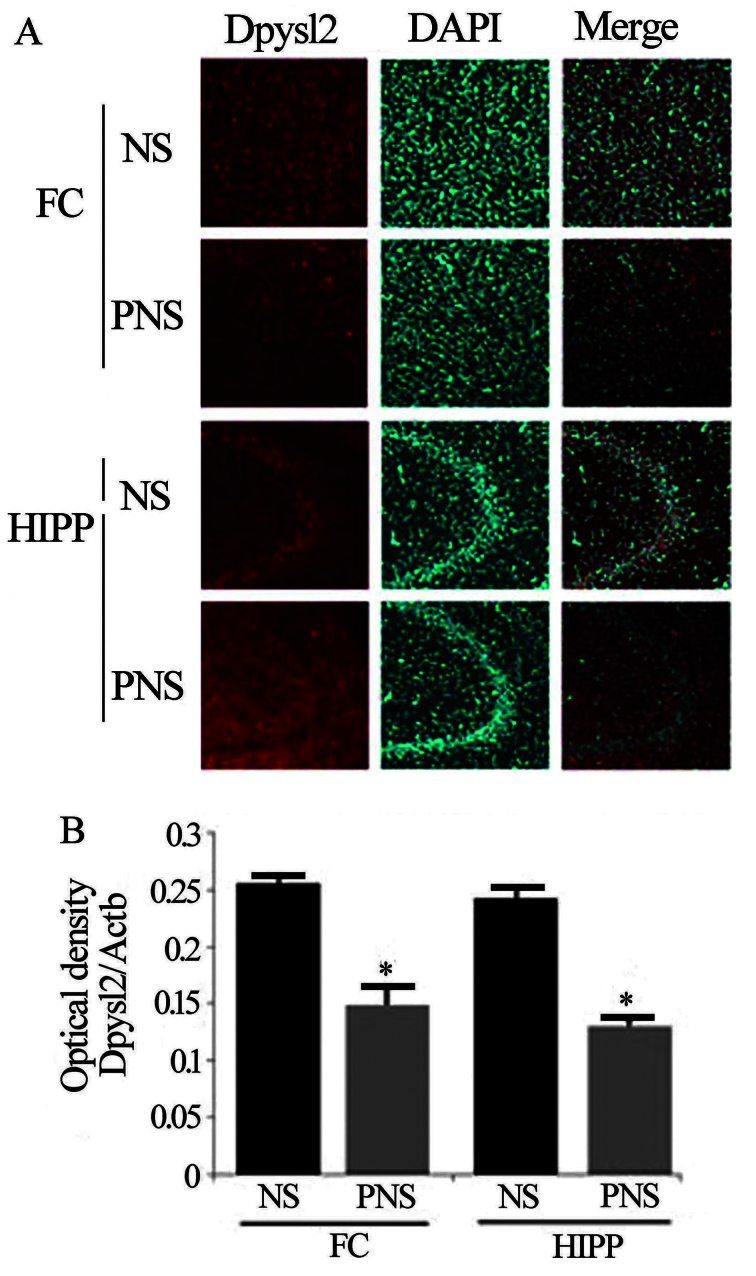
Immunohistochemical analysis of Dpysl2 expression in the brains of PNS-induced rats. (A) Confocal microscopic image showing immunofluorescent staining for Dpysl2 (anti-Dpysl2, red, Cy3) with DAPI in the prefrontal cortex and hippocampus. Fluorescent staining revealed decreased Dpysl2 in these regions. Scale bar, FC; 50 Hipp; 100 *μ*m. (B) Optical densities of Dpysl2 signals in immunostained prefrontal cotex and hippocampus sections from (^*^P<0.05 compared with the NS group in the prefrontal cortex and hippocampus). Dpysl2, dihydropyrimidinase-like 2; FC, prefrontal cortex; Hipp, hippocampus; NS, non-prenatal stressed offspring; PNS, prenatal stressed offspring; SD, standard deviation.

Dpysl2 is expressed in neurons of the central nervous system and is concentrated at synaptic sites and in the axon, where it may affect synaptic physiology (34). To confirm the PNS-induced downregulation of Dpysl2 and Dpysl3 proteins observed in our proteomics study (Fig. 3), we performed western blotting (Figs. 4 and 5) and immunohistochemical (Fig. 6) analyses of the hippocampal and prefrontal cortex areas of the control and PNS rat brains. In the western blot analyses used to assess the Dpysl2 and Dpysl3 proteins, 100 and 20 *μ*g of the lysed protein, respectively, were electrophoresed on a 10% SDS-PAGE gel and then transferred onto a PVDF membrane. The membranes were probed with anti-Dpysl2, anti-Dpysl3 and anti-Actb antibody overnight and then with peroxidase-conjugated secondary antibodies prior to immu-noreactive bands being detected using the ECL kit. Western blot analyses revealed that the amount of Dpysl2 protein in the prefrontal cortex and hippocampus was significantly lower in PNS than in NS rats (all P-values <0.05) (Fig. 4), whereas the amount of Actb in the prefrontal cortex and hippocampus was similar in the two groups. Similar results were observed for Dpysl3 (all P-values <0.05 in the prefrontal cortex and hippocampus) (Fig. 5).

### Immunohistochemical analysis

To detect Dpysl2 expression, the fixed brains were removed, frozen, and cut into 30-*μ*m sections. The frozen sections from the rat prefrontal cortices and hippocampi were incubated with individual antibodies of Dpysl2 and then incubated with a Cy3-conjugated anti-rabbit secondary antibody. The differential expression of the Dpysl2 proteins was evident in the immunofluorescence-stained images of NS and PNS rat brains and in the intensity measurements of the immunohistochemical staining of Dpysl2 in these images (P<0.001 for the prefrontal cortex and the hippocampus in Dpysl2) (Fig. 6).

### Genetic association of DPYSL2 SNPs with schizophrenia in a Korean population

After identification of Dpysl2 as a PNS-induced protein in the rat, the study was extended to human subjects. To evaluate whether genetic polymorphisms of the human *DPYSL2* gene are associated with schizophrenia, seven SNPs (rs9886448, rs4872449, rs431246, rs2289593, rs327222, rs708621 and rs17666) were selected partially based on the identification of five SNPs (35) and two promoter SNPs of the *DPYSL2* that may have affected gene expression in a previously described Japanese population. The genotyping of six SNPs was performed by enzyme digestion of the post-PCR product and by the HRM method for one SNP. The genotypic and allelic distributions of the SNPs in patients and control subjects are presented in Table VII. The genotype distributions of the seven SNPs were analyzed using HWE (P>0.05).

The heterozygous genotype (TC) frequency for rs9886448 was lower in the schizophrenia group (27.20%) than in the control group (41.64%), and its genotypic frequency was statistically associated with schizophrenia in codominant models 1 and 2 [OR, 0.54 (1.20); 95% CI, 0.36–0.80 (0.66–2.17); P=0.003, P-value corrected by the Bonferroni method, Pc=0.020] and in the overdominant model (OR, 0.52; 95% CI, 0.36–0.77; P=0.001, Pc=0.006). Furthermore, the allele frequency for rs2289593 was lower in the schizophrenia group (7.67%) compared with the control group (14.2%), and its genotypic frequency was statistically associated with schizophrenia in codominant models 1 and 2 [OR, 0.48 (0.29); 95% CI, 0.29–0.79 (0.06–1.37); P=0.003, Pc=0.023], the dominant model (OR, 0.46; 95% CI, 0.29–0.74; P=0.001, Pc=0.006), the overdominant model (OR, 0.49; 95% CI, 0.30–0.81; P=0.004, Pc=0.026), and the log-additive model (OR, 0.49; 95% CI, 0.32–0.76; P=0.001, Pc=0.006). The allele frequency of rs2289593 was also associated with susceptibility to schizophrenia (OR, 0.50; 95% CI, 0.33–0.77; P=0.002, Pc=0.014) (Table VII). No significant differences were found between patients and control subjects regarding the frequency of the genotype or allele in five of the seven polymorphisms i.e., rs4872449, rs431246, rs327222, rs708621 and rs17666.

The haplotype of the polymorphisms within the *DPYSL2* gene was evaluated using the Haploview and SNPstats programs. Of the seven SNPs, no LD blocks were constructed using the Gabriel method (38; data not shown). The D’ values between the SNPs were <0.5, which indicates a very weak LD between each pair of markers. As none of the LD blocks constructed included the three SNPs, a haplotype association study was not performed. The power of the sample size was calculated using a genetic power calculator (http://pngu.mgh.harvard.edu/~purcell/gpc), and the experimental error was reduced by an adjusted effective sample size (calculated sample size ×100/95). When the sample power (α=0.05, relative risk =2, prevalence =0.02) was estimated, the case-control study was sufficiently powerful to determine a positive association. In this study, it was 0.9144 for rs9886448 (number of cases for 80% power, n=143), 0.9161 for rs4872449 (n=142), 0.9323 for rs431246 (n=132), 0.9387 for rs2289593 (n=129), 0.944 for rs327222 (n=125), 0.9308 for rs708621 (n=133), and 0.9409 for rs17666 (n=127).

### Promoter activity of SNPs located in the promoter region

We investigated whether the two promoter SNPs (rs9886448 and rs4872449) altered the transcriptional activity of the *DPYSL2* promoter sequence. The results of an *in silico* promoter-binding prediction algorithm (http://www.gene-regulation.com/pub/programs/alibaba2) suggested that SNPs may not affect *DPYSL2* gene transcription (data not shown) and, thus, their effects on promoter activity in human SH-SY5Y neuroblastoma cells was examined *in vitro*. To assess the promoter activity of the SNPs, plasmids containing one allele from each SNP and a luciferase reporter gene were transfected into human SH-SY5Y neuroblastoma cells, and the reporter activities of the four constructs of the two SNPs were compared. No significant differences in luciferase activity was observed in any of the pGL3 basic constructs with alleles of the SNPs (relative promoter activity in percentage): rs9886448; pGL3-allele/pRL-SV40, pGL3-basic, 100.00±37%; TT, 57.92±22%; CC, 74.21±25%; and rs4872449; pGL3-basic, 100.00±19%; AA, 104.61±35%; GG, 93.82±28%.

## Discussion

The present study investigated a novel target, *DPYSL2*, to examine the pathophysiology of schizophrenia using proteomic analyses in a PNS animal model associated with the neurodevelopmental theory of schizophrenia.

Dpysl2, also known as collapsin response mediator protein 2 (Crmp2), was first identified in chick dorsal root ganglia cultures as a signal transducer responsible for axon growth cone retraction induced by negative guidance signals from the semaphorin 3A (Sema3A) pathway of the developing nervous system (37). Dpysl2 is a multifunctional adaptor protein in the central nervous system that uses a cytosolic protein as the primary sequence homology to the dihydropyrimidinase enzyme (DPYS) responsible for uracil and thymine catabolism (39). In the developing brain, Dpysl2 regulates axonal outgrowth via the promotion of microtubule assembly, vesicle trafficking, and synaptic physiology (15,31–43). In experiments investigating Dpysl2 and its derivatives, only the C-terminal region mediates microtubule binding, and Dpysl2-depleted cells exhibit destabilized anaphase astral microtubules and altered spindle positions.

An 82-residue C-terminal region of Dpysl2, unrelated to other microtubule binding motifs, is sufficient to stabilize microtubules (41), and Dpysl2 has been shown to be involved in the regulation of neurite outgrowth in the neurite shaft and growth cone (42). Dpysl2 binds directly to N-type Ca^2+^ (CaV2.2) channels in two regions: the channel domain I–II intracellular loop and the distal C terminus (43). Overexpression of the Dpysl2 protein in hippocampal neurons causes an increase in Ca^2+^ channel present density whereas a lentivirus-mediated Dpysl2 knockdown eliminates this effect (43). The study also revealed an increased number of CaV2.2 channels on the cell surface of Dpysl2-overexpressing neurons, which also show a significant increase in vesicular release in response to a depolarizing stimulus. The depolarization of Dpysl2-overexpressing neurons elicits an increase in the release of glutamate (43), and dysfunction within the Dpysl2 system may result in neuro developmental abnormalities, such as unregulated axonal growth and branching, which may be a factor into the pathogenesis of schizophrenia. The expression of *DPYSL2* in humans has been reported to be decreased in the brains of patients with schizophrenia (44). Additionally, *DPYSL2* is located on chromosome 8p21, a region that has been associated with schizophrenia in genetic linkage studies (45).

The present findings suggest that application of a repeated variable PNS paradigm during the critical periods of fetal brain development results in Dpysl2 protein expression changes that may have enduring effects on axonal outgrowth and synaptic function in the offspring during adulthood. Taken together with functional positional evidence, the present experimental results indicate that mutations or polymorphisms in and/or nearby the *DPYSL2* gene may play a role in genetic susceptibility for and development of schizophrenia. Thus, the association between the functional SNPs of *DPYSL2* and schizophrenia was examined. The present findings demonstrated that SNPs in the promoter (rs9886448) and exon (rs2289593) regions were associated with susceptibility for schizophrenia. Polymorphisms of the promoter SNP (rs9886448) did not affect the promoter activity of the sequence around the SNP. However, the functional alterations that may have been induced by these polymorphisms (rs2289593) have not been evaluated, which is a limitation of this study,

The genotype frequencies for SNPs in Korean, European, Chinese, Japanese, and Sub-Saharan African populations were compared to confirm the present genotyping data (Table VIII). The rs9886448, rs4872449, rs2289539 and rs708621 genotype distributions in the present control Korean group were generally similar to those of the Chinese and Japanese populations. However, the A/G genotype of rs2289539 has not been detected in any population, although the A/G genotype in the present population was detected at high frequencies. The rs327222 genotype distribution was similar to the Sub-Saharan African distribution and the rs17666 genotype distribution was similar to the European distribution. However, the genotype distributions of the European, Chinese, Japanese and Sub-Saharan African populations were generally not similar to that of the present schizophrenia group. Therefore, the genotypic differences among ethnic groups may be derived from the ethnicity of each population, and the genotype distributions of the present schizophrenia group may have been associated with the disease condition. These comparisons suggest that the present genotyping results are reliable for genetic association studies as most of the distributions were similar to the Asian population albeit not to the European and Sub-Saharan African populations.

Additionally, the candidate gene sequences from humans were compared with those of mice to identify a direct relationship between the human *DPYSL2* gene and polymorphisms in mice (data not shown). The mRNA sequence of the gene was very similar across species (http://blast.ncbi.nlm.nih.gov); the maximum identity of the gene was 87%, and the transcript sequences of the human gene were very similar to those of mice. However, the exonic SNP of the human gene was not identified in rat sequences. Although the site is the same in the two species, it is not polymorphic in the rat. A polymorphism of the human *DPYSL2* gene located in the promoter region (rs9886448) was not identified in the rat sequences as the promoter region of human and mouse sequences differ. Therefore, none of the polymorphisms of the human *DPYSL2* was identified in the rat genome, which is another limitation of the present study.

In this study, *DPYSL2* was shown to be affected by the PNS paradigm and was associated with the neurodevelopmental theory of schizophrenia. PNS during gestation is involved in the pathology of various psychiatric disorders, such as schizophrenia and depression, and constitutes a pathogenetic theory. Based on gel-based proteomic and genetic population studies, PNS during the critical periods of fetal brain development can result in clearly altered expression patterns of Dpysl2. The upstream and missense SNPs of the PNS-associated gene *DPYSL2* exhibit clear alterations in genetic studies of human patients with schizophrenia. These results suggest that *DPYSL2* is likely associated with susceptibility to schizophrenia in humans, which is consistent with the association of Dpysl2 changes in the rat model of PNS.

In conclusion, the neurodevelopmental theory of schizophrenia and susceptibility to schizophrenia appear to connect the *DPYSL2* gene with the disease as the present results suggest *DPYSL2* and its SNPs in the pathophysiology of schizophrenia. To the best of our knowledge, this is not the first study to identify a relationship between the *DPYSL2* gene and schizophrenia, but it is the first to investigate this relationship in terms of the neurodevelopmental theory of schizophrenia. Additionally, this study investigates the correlation in a Korean population. Therefore, the present study provides valuable data regarding pathogenesis of psychiatric disorders such as schizophrenia. However, studies using cellular or animal model systems are required to elucidate the actual role of *DPYSL2*.

## Figures and Tables

**Table I:** Polymerase chain reaction primers for single-nucleotide polymorphisms in DPYSL2.

SNP	Primer sequences	Annealing temperature (°C)	Product size (bp)	Restriction enzyme
rs9886448 (−1625T>C)	F: 5′-GAAGCCAGACTTTGAGGTGGA-3′ R: 5′-GCACTGCTTTCCAGGTTAGG-3′	58	120	N/A
rs4872449 (−1195A>G)	F: 5′-CCGCACTTACTCCCCAAACA-3′ R: 5′-ACGTGTCGCGATTTGGTTTA-3′	52	317	*Sma*I
rs431246 (−975C>G)	F: 5′-GCCCATTCCCCGCCCCCAGGAG-3′ R: 5′-CCCGCCGTTGCTGGCGCTGAAC-3′	70	169	*Alw*62I
rs2289593 (352G>A)	F: 5′-ACCTACCGTGATCCTTCACAAG-3′ R: 5′-AGCTGGGTTACATGGATTCTTA-3′	53	327	*Fnu*4HI
rs327222 (426C>T)	F: 5′-ACTACTCTCTGCATGTGGACATCCG-3′ R: 5′-GAGGGCATTGACTCAGTGCAACCTA-3′	62	104	*Bsh*1236I
rs708621 (1506T>C)	F: 5′-GCTGAGCTGAGAGGCGTTCCTC-3′ R: 5′-CCTGCTGCTTGGCAGGAGACGT-3′	56	118	*Bsp*LI
rs17666 (*2236T>C)	F: 5′-GTCTTCCTGTTTTTCCTGTACC-3′ R: 5′-TATTTTGCCATCAAGACAGTGG-3′	56	367	*Bsp*TI

N/A, not applicable; SNP, single-nucleotide polymorphisms; DPYSL2, dihydropyrimidinase-like 2; F, forward; R, reverse.

**Table II:** Social interaction behavior of non-prenatal stress and prenatal stress-induced rat.

Behavior	NS	PNS	P-value
Non-aggressive			
Sniffing (n)	18.4±2.26	17.1±2.79	0.722
Sniffing (s)	74.7±13.62	65.7±11.24	0.617
Following (n)	14±2.29	9.62±2.13	0.059
Following (s)	55.6±9.24	41.37±8.72	0.095
Grooming the partner (n)	2.2±0.69	0.29±0.18	**0.011**
Grooming the partner (s)	16±7.73	5.43±4.64	0.164
Aggressive			
Fight (n)	5±1.14	3.16±1.4	0.173
Fight (s)	20±9.08	19.16±8.47	0.645
Aggressive grooming (n)	1±0.00	1±0.00	1.000
Aggressive grooming (s)	4±0.00	3±0.00	0.844
Biting (n)	2.5±1.50	4±0.00	0.104
Biting (s)	14.5±9.50	16±0.00	0.112

Bold, significant decrease. Data are presented as mean ± SEM; n, number of the behavior; s, duration measured in seconds; NS, non-stressed group; PNS, prenatally-stressed group; SEM, standard error of the mean.

**Table III:** Behavior of non-prenatal stress and prenatal stress induced rat in an open field.

Behavior	NS	PNS	P-value
Centrally entered (n)	5.1±1.87	1.3±0.57	0.068
Line crossing (n)	1.8±0.46	0.4±0.22	0.014
Run (n)	15.8±3.69	17.4±0.37	0.753
Run (s)	19±4.32	26±5.96	0.316
Rear (n)	44.2±7.81	35.6±3.26	0.323
Rear (s)	103.2±24.90	82.4±13.36	0.471
Grooming (n)	13.7±1.14	14.4±0.81	0.625
Grooming (s)	283.3±34.15	283.4±24.41	0.998
Cage sniff (n)	22.3±2.35	25.8±4.27	0.482
Cage sniff (s)	62.3±7.94	84.3±18.32	0.285
Immobile (n)	7.8±1.83	12.1±2.07	0.139
Immobile (s)	208.9±55.06	263.8±47.39	0.460

Data are presented as mean ± SEM. n, number of the behavior; s, duration measured in seconds; NS, non-stressed group; PNS, prenatally-stressed group; SEM, standard error of the mean.

**Table IV:** Behavioral response in the forced swim test session.

Behavior	NS	PNS	P-value
Climbing	75±7.56	81±10.87	0.656
Swimming	103±5.06	74±11.12	0.029
Immobility	122±6.79	144±15.08	0.191

Data are presented as mean ± SEM. NS, non-stressed group; PNS, prenatally-stressed group; SEM, standard error of the mean.

**Table V:** A list of prefrontal cortex proteins that are differentially expressed in non-prenatal stress and prenatal stress-induced rats.

Protein description	Access no.	Nominal mass	pI	Score	Fold-change^c^	Coverage (%)
Upregulated in prenatal stress induced rat						
Phosphoglycerate mutase 1	gi|114326546	28928	6.67	88	>200	55
Protein carboxyl methyltransferase	gi|603467	24667	7.14	61	200	29
GTP-binding nuclear protein Ran	gi|5453555	24579	7.01	113	200	55
p55 protein	gi|83320109	51317	6.01	62	2.3	29
Proteasome subunit α type-2	gi|8394063	26024	6.92	87	1.3	38
Downregulated in prenatal stress induced rat						
Dihydropyrimidinase-like 2	gi|40254595	62638	5.95	233	0.7	48
Septin-6	gi|290677865	49147	6.23	191	0.7	38
Dihydropyrimidinase-like 3	gi|25742568	62327	6.04	114	0.7	31
Vinculin	gi|157822133	117112	5.83	223	0.7	28
Glycerol-3-phosphate dehydrogenase [NAD^+^], cytoplasmic	gi|57527919	38112	6.16	151	0.7	48
Dihydropyrimidinase-like 2	gi|40254595	62638	5.95	102	0.6	26
Dihydropyrimidinase-like 2	gi|40254595	62638	5.95	178	0.6	44
Dihydropyrimidinase-like 3, isoform CRA^b^	gi|149017448	62141	6.04	104	0.6	28
Gelsolin, isoform CRA^b^	gi|149038929	81064	5.46	92	0.58	22
Dihydropyrimidinase-like 2	gi|40254595	62638	5.95	150	0.56	36
Inner membrane protein, mitochondrial, isoform CRA^a^	gi|149036390	86204	5.67	124	0.55	26
NADH dehydrogenase (ubiquinone) Fe-S protein 1, isoform CRA^b^	gi|149046009	74362	5.74	229	0.49	35
Transitional endoplasmic reticulum ATPase	gi|17865351	89977	5.14	227	0.42	37
Transitional endoplasmic reticulum ATPase	gi|17865351	89977	5.14	206	0.41	32
Ubiquitin carboxylterminal hydrolase 14	gi|56605688	56397	5.11	65	0.4	22
Inner membrane protein, mitochondrial, isoform CRA^a^	gi|149036390	86204	5.67	196	0.4	31
α-internexin	gi|55622	55712	5.2	327	0.4	47
Glial fibrillary acidic protein δ	gi|5030428	48809	5.72	145	0.4	36
Glial fibrillary acidic protein	gi|430721	49970	5.35	141	0.4	36
Coiled-coil domain containing 85A-like isoform 3	gi|293341895	50812	8.29	64	0.3	16
β-soluble NSF attachment protein	gi|205829956	33791	5.32	233	0.29	75
α-soluble NSF attachment protein	gi|18034791	33627	5.3	236	0.2	73
Tpi1 protein	gi|38512111	27214	7.07	187	0.2	65
Dynactin subunit 2	gi|51948450	44235	5.14	251	0.2	50
Sirtuin (silent mating type information regulation 2 homolog) 2 (*S. cerevisiae*), isoform CRA^a^	gi|149056443	43763	5.37	91	0.2	34
Serum albumin precursor	gi|158138568	70710	6.09	284	0.2	49

a Factor by which spot differs in total intensity from the corresponding spot for the non-prenatal stress sample.

b Percentage of amino-acid sequence covered by peptides in the MALDI-TOF MS analysis.

**Table VI:** A list of hippocampal proteins that are differentially expressed in non-prenatal stress and prenatal stress-induced rats.

Protein description	Access no.	Nominal mass	pI	Score	Fold-change^c^	Coverage (%)
Upregulated in prenatal stress induced rat						
NADH dehydrogenase (ubiquinone) Fe-S protein 1, isoform CRA^b^	gi|149046009	74362	5.74	229	1.23	35
Vinculin	gi|157822133	117112	5.83	223	1.2	28
p55 protein	gi|83320109	51317	6.01	62	1.1	29
α-internexin	gi|55622	55712	5.2	327	1.1	47
β-soluble NSF attachment protein	gi|205829956	33791	5.32	233	1.07	75
Downregulated in prenatal stress induced rat						
Dihydropyrimidinase-like 2	gi|40254595	62638	5.95	150	0.7	36
Dihydropyrimidinase-like 2	gi|40254595	62638	5.95	233	0.7	48
Tpi1 protein	gi|38512111	27214	7.07	187	0.7	65
Dynamin-1	gi|190358918	97576	6.44	264	0.7	38
Hydroxyacylglutathione hydrolase, mitochondrial precursor	gi|315630402	34593	8.06	78	0.7	34
Actin-related protein 2/3	gi|205686193	34484	6.84	164	0.6	41
Electron transfer flavoprotein subunit α, mitochondrial precursor	gi|57527204	35272	8.62	146	0.6	38
Glycerol-3-phosphate dehydrogenase [NAD^+^], cytoplasmic	gi|57527919	38112	6.16	151	0.6	48
Glycogen phosphorylase, brain form	gi|158187544	97361	6.31	125	0.6	14
Transitional endoplasmic reticulum ATPase	gi|17865351	89977	5.14	206	0.57	32
Protein carboxyl methyltransferase	gi|603467	24667	7.14	61	0.5	29
Proteasome subunit α type-2	gi|8394063	26024	6.92	87	0.5	38
N-acetylneuraminic acid synthase	gi|164663874	40482	6.39	82	0.5	21
Ribose-phosphate pyrophosphokinase 1 isoform 1	gi|4506127	35325	6.51	85	0.5	24
Glycogen phosphorylase, brain form	gi|158187544	97361	6.31	68	0.5	10
Septin-6	gi|290677865	49147	6.23	191	0.5	38
Dihydropyrimidinase-like 3	gi|25742568	62327	6.04	114	0.5	31
Sirtuin (silent mating type information regulation 2 homolog) 2 (*S. cerevisiae*), isoform CRA^a^	gi|149056443	43763	5.37	91	0.47	34
GTP-binding nuclear protein Ran	gi|5453555	24579	7.01	113	0.4	55
Hydroxyacylglutathione hydrolase, mitochondrial precursor	gi|315630402	34593	8.06	76	0.4	30
Ubiquitin carboxyl-terminal hydrolase 14	gi|56605688	56397	5.11	65	0.4	22
α-soluble NSF attachment protein	gi|18034791	33627	5.3	236	0.4	73
Coiled-coil domain containing 85A-like isoform 3	gi|293341895	50812	8.29	64	0.3	16
Dihydropyrimidinase-like 2	gi|40254595	62638	5.95	178	0.3	44
Dynactin subunit 2	gi|51948450	44235	5.14	251	0.3	50

a Factor by which spot differs in total intensity from the corresponding spot for the non-prenatal stress sample.

b Percentage of amino-acid sequence covered by peptides in the MALDI-TOF MS analysis.

**Table VII:** Association between frequency of DPYSL2 polymorphisms and schizophrenia.

SNP	Genotype/allele	Control		Schizophrenia		Model	OR (95% CI)	P-value	Pc-value
		n	%	n	%				
rs9886448 (promoter-1625T>C)	TT	158	49.84	122	60.4	Codominant 1	0.54 (0.36–0.80)	0.003	0.02
	TC	132	41.64	55	27.2	Codominant 2	1.20 (0.66–2.17)		
	CC	27	8.52	25	12.4	Dominant	0.65 (0.46–0.93)	0.018	0.126
						Recessive	1.52 (0.85–2.70)	0.16	1
						Overdominant	0.52 (0.36–0.77)	0.001	0.006
						Log-additive	0.86 (0.66–1.12)	0.26	1
	T	448	70.7	299	74.0				
	C	186	29.3	105	26.0		0.846 (0.639–1.120)	0.242	1
rs4872449 (promoter-1195A>G)	AA	149	48.85	93	46	Codominant 1	0.99 (0.68–1.44)	0.008	0.056
	AG	135	44.26	83	41.1	Codominant 2	1.98 (1.06–3.73)		
	GG	21	6.89	26	12.9	Dominant	1.12 (0.78–1.60)	0.53	3.71
						Recessive	2.00 (1.09–3.66)	0.024	0.168
						Overdominant	0.88 (0.61–1.26)	0.48	1
						Log-additive	1.23 (0.94–1.62)	0.14	0.98
	A	433	71.0	269	66.6				
	G	177	29.0	135	33.4		1.228 (1.609–0.936)	0.138	0.966
rs431246 (promoter-975C>G)	CC	176	55.5	117	57.9	Codominant 1	0.73 (0.49–1.07)	0.011	0.077
	CG	120	37.9	58	28.7	Codominant 2	1.93 (1.04–3.58)		
	GG	21	6.6	27	13.4	Dominant	0.91 (0.63–1.30)	0.59	1
						Recessive	2.17 (1.19–3.96)	0.011	0.077
						Overdominant	0.66 (0.45–0.97)	0.031	0.217
						Log-additive	1.10 (0.85–1.44)	0.47	1
	C	472	74.5	292	72.3				
	G	162	25.5	112	27.7		1.118 (0.843–1.481)	0.439	1
rs2289593 (missense 352G>A)	GG	236	74.45	173	85.64	Codominant 1	0.48 (0.29–0.79)	0.003	0.023
	GA	72	22.71	27	13.37	Codominant 2	0.29 (0.06–1.37)		
	AA	9	2.84	2	0.99	Dominant	0.46 (0.29–0.74)	0.001	0.006
						Recessive	0.33 (0.07–1.58)	0.13	0.91
						Overdominant	0.49 (0.30–0.81)	0.004	0.026
						Log-additive	0.49 (0.32–0.76)	0.001	0.006
	G	544	85.80	373	92.33				
	A	90	14.20	31	7.67		0.502 (0.327–0.771)	0.002	0.014
rs327222 (synonymous 426C>T)	CC	226	71.29	151	74.75	Codominant 1	0.95 (0.63–1.45)	0.073	0.511
	CT	78	24.61	49	24.26	Codominant 2	0.22 (0.05–1.00)		
	TT	13	4.10	2	0.99	Dominant	0.84 (0.56–1.27)	0.14	0.98
						Recessive	0.22 (0.05–1.01)	0.023	0.161
						Overdominant	0.99 (0.66–1.51)	0.98	1
						Log-additive	0.78 (0.55–1.11)	0.16	1
	C	530	83.60	351	86.88				
	T	104	16.40	53	13.12		0.770 (0.538–1.100)	0.151	1
rs708621 (synonymous 1506T>C)	TT	181	57.10	112	55.45	Codominant 1	1.09 (0.74–1.60)	0.86	1
	CT	108	34.07	74	36.63	Codominant 2	0.93 (0.48–1.81)		
	CC	28	8.83	16	7.92	Dominant	1.06 (0.74–1.52)	0.76	1
						Recessive	0.90 (0.47–1.72)	0.74	1
						Overdominant	1.10 (0.76–1.60)	0.61	1
						Log-additive	1.01 (0.77–1.34)	0.92	1
	C	470	74.13	298	73.76				
	T	164	25.87	106	26.24		1.019 (0.767–1.354)	0.895	1
rs17666 (3′UTR *2236T>C)	TT	234	73.8	161	79.7	Codominant 1	0.81 (0.52–1.25)	0.03	0.21
	TC	72	22.7	40	19.8	Codominant 2	0.13 (0.02–1.03)		
	CC	11	3.5	1	0.5	Dominant	0.72 (0.47–1.10)	0.12	0.84
						Recessive	0.14 (0.02–1.08)	0.015	0.105
						Overdominant	0.85 (0.55–1.32)	0.48	1
						Log-additive	0.68 (0.47–1.01)	0.049	0.343
	T	540	85.1	362	89.6				
	C	94	14.9	42	10.4		0.667 (0.452–0.982)	0.04	0.28

OR, odds ratio; CI, confidence interval; Pc-value, Bonferroni corrected P-value. DPYSL2, dihydropyrimidinase-like 2.

**Table VIII:** Genotype frequencies of *DPYSL2* SNPs in various populations (www.ncbi.nlm.nih.gov/SNP, dbSNP Build 139).

SNP	Genotype/allele	Korean		European	Chinese	Japanese	Sub-Saharan African
		Schizophrenia	Control				
rs9886448	C/C	0.085	0.124	0.018	0.047	0.07	0.035
	C/T	0.416	0.272	0.15	0.326	0.384	0.292
	T/T	0.498	0.604	0.832	0.628	0.547	0.673
	C	0.26	0.293	0.093	0.209	0.262	0.181
	T	0.74	0.707	0.907	0.791	0.738	0.819
rs4872449	A/A	0.46	0.489	0.203	0.4	0.432	0
	A/G	0.411	0.443	0.508	0.489	0.409	0
	G/G	0.129	0.069	0.288	0.111	0.159	1
	A	0.666	0.71	0.458	0.664	0.636	0
	G	0.334	0.29	0.542	0.356	0.364	1
rs2228979	A/A	0.01	0.028	0	0.133	0.114	0
	A/G	0.134	0.227	0	0	0	0
	G/G	0.856	0.745	1	0.867	0.886	1
	A	0.077	0.142	0	0.067	0.057	0
	G	0.923	0.858	1	0.933	0.943	1
rs327222	C/C	0.748	0.713	0.991	0.814	0.895	0.761
	C/T	0.243	0.246	0.009	0.186	0.105	0.221
	T/T	0.01	0.041	0	0	0	0.018
	C	0.869	0.836	0.996	0.907	0.948	0.872
	T	0.131	0.164	0.004	0.093	0.052	0.128
rs708621	C/C	0.079	0.088	0.55	0.113	0.133	0.65
	C/T	0.366	0.341	0.367	0.556	0.378	0.3
	T/T	0.555	0.571	0.083	0.311	0.489	0.05
	C	0.738	0.741	0.773	0.411	0.322	0.8
	T	0.262	0.259	0.267	0.589	0.678	0.2
rs17666	C/C	0.005	0.035	0.023	0.012	0.239	0.306
	C/T	0.198	0.227	0.279	0.314	0.451	0.449
	T/T	0.797	0.738	0.689	0.674	0.31	0.245
	C	0.104	0.149	0.163	0.169	0.465	0.531
	T	0.896	0.851	0.837	0.831	0.535	0.469

DPYSL2, dihydropyrimidinase-like 2; SNP, single nucleotide polymorphism.
